# Use of new radiochromic devices for peripheral dose measurement: potential in-vivo dosimetry application

**DOI:** 10.2349/biij.5.4.e16

**Published:** 2009-10-01

**Authors:** S-T Chiu-Tsao, MF Chan

**Affiliations:** 1 Quality MediPhys LLC, Denville, New Jersey, United States; 2 Memorial Sloan-Kettering Cancer Center, Basking Ridge, New Jersey, United States

**Keywords:** Peripheral dose, radiochromic film, critical structures, in-vivo dosimetry

## Abstract

The authors have studied the feasibility of using three new high-sensitivity radiochromic devices in measuring the doses to peripheral points outside the primary megavoltage photon beams. The three devices were GAFCHROMIC® EBT film, prototype Low Dose (LD) Film, and prototype LD Card. The authors performed point dosimetry using these three devices in water-equivalent solid phantoms at x = 3,5,8,10, and 15 cm from the edge of 6 MV and 15 MV photon beams of 10x10 cm^2^, and at depths of 0, 0.5 cm, and depth of maximum dose. A full sheet of EBT film was exposed with 5000 MU. The prototype LD film pieces were 1.5x2 cm^2^ in size. Some LD films were provided in the form of a card in 1.8x5 cm^2^ holding an active film in 1.8x2 cm^2^. These are referred to as “LD dosimeter cards”. The small LD films and cards were exposed with 500 MU. For each scanned film, a 6 mm circular area centered at the measurement point was sampled and the mean pixel value was obtained. The calibration curves were established from the calibration data for each combination of film/cards and densitometer/scanner. The doses at the peripheral points determined from the films were compared with those obtained using ion chamber at respective locations in a water phantom and general agreements were found. It is feasible to accurately measure peripheral doses of megavoltage photon beams using the new high-sensitivity radiochromic devices. This near real-time and inexpensive method can be applied in a clinical setting for dose measurements to critical organs and sensitive patient implant devices.

## INTRODUCTION

In the delivery of radiation treatments, it is important to monitor the peripheral doses to critical organs, like gonad and fetus [1], and sensitive implant devices, such as pacemaker and intracardial defibrillator (ICD). Current methods include ion chamber [2,3], thermoluminescent dosimetry (TLD) [4,5], diode [6,7], MOSFET [8,9], optically stimulated luminescent dosimeters (OSLD) [10,11], and Monte Carlo modeling [12-15]. In this study, the authors explored the possibility of using new high-sensitivity radiochromic films (RCF) to measure the peripheral doses.

There have been many reports on radiochromic film as a quantitative two-dimensional (2D) dosimeter with fine spatial resolution [16-33]. There are many different models of radiochromic films produced by the International Specialty Products (ISP), under the trade name Gafchromic®. Since its introduction in 2004, Gafchromic® EBT film has emerged as a strong candidate for 2D dosimetry [19, 21-30] in the clinical dose range and for the radiation field with heterogeneous energy spectrum, due to the high sensitivity [19,34], weak energy dependence [25,27,34,35], and tissue equivalence [30] (Z_eff_ = 6.98). Compared with the early models, EBT films have improved film uniformity [30]. EBT film has been used for skin dosimetry at the air interface for conventional and IMRT modalities for quite some time [36-40].

Recently introduced was the new prototype model of RCF, referred to as the low dose (LD) film (ISP, Wayne, NJ, USA), which has higher sensitivity compared with the EBT films by about a factor of 10, and a similar active emulsion material as the EBT film. While the EBT film has application for doses up to 800 cGy or more, the LD film has been designed to be especially useful for the dose range from 1 to 40 cGy. With a similar active emulsion material as the EBT film, the dose response of the LD film is also expected to have weak energy dependence. In addition, the sensitivity of the LD film makes it suitable for the dosimetry in the clinical dose range for the peripheral region of megavoltage photon field. Some LD films were provided in the form of a card about 1.8×5 cm^2^ in size holding a piece of the active film about 1.8×2 cm^2^. These were referred to as “LD dosimeter cards”.

In this work, the authors studied the feasibility of using three high-sensitivity radiochromic devices, in measuring the doses to peripheral points outside the primary photon beams at the air interface and d = 0.5 cm as well as d_max_ (1.5 cm and 3 cm) in a water-equivalent solid phantom for 6 MV and 15 MV photon beams. The film dose measurements were made at five locations in the peripheral region at the distances from the field edge of 3 cm to 15 cm. The three devices were GAFCHROMIC® EBT film, prototype LD Film, and prototype LD Card. For comparison, ion chamber measurements were also carried out at the same peripheral locations.

## MATERIALS AND METHOD

### Radiochromic films

**Figure 1:**
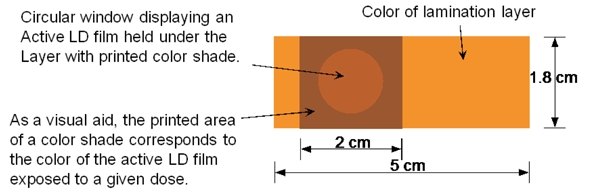
Diagram of the prototype LD dosimeter card used in this study.

The authors performed point dosimetry using three radiochromic devices in water-equivalent solid phantoms at x = 3, 5, 8, 10, and 15 cm from the edge of a 10×10 cm^2^ field of a 6 MV and a 15 MV beam on a Varian Clinac-iX, and at the depths of 0, 0.5 cm and d_max_, 100 cm SAD. The EBT film lot#35076 and prototype LD film lot#36263 from ISP were used in this study. Each LD film, cut to a size of 1.5×2 cm^2^, was used as a point detector for dose measurement. Some LD films were provided in the form of a card about 1.8×5 cm^2^ in size holding a piece of active film about 1.8×2 cm^2^, referred to as “LD dosimeter card”, as shown in Figure 1. Each LD card has a circular window displaying an active LD film held under a yellow lamination layer with printed color shade surrounding the circular window. The circular window area was designed for point-dose measurement. The thicknesses of the EBT film, LD film, and LD card were 0.24 mm, 0.6 mm, and 1.1 mm, respectively.

### Irradiation of calibration films

**Figure 2:**
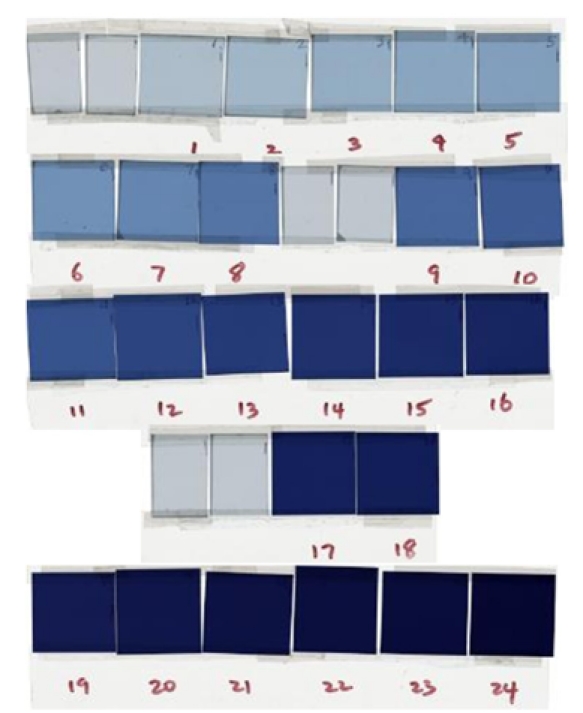
Scanned images of the calibration EBT films obtained in this study. The doses to the calibration films ranged from 20 (film #1) to 5000 cGy (film #24). The films without number label were background films kept in this set.

**Figure 3:**
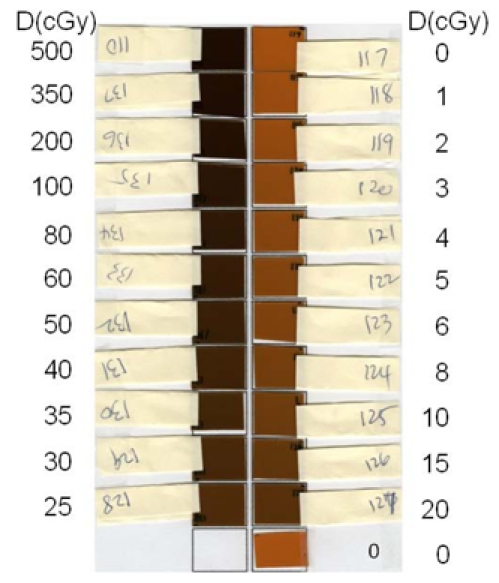
Image of the calibration and background LD films scanned together in this study. The light yellow paper piece attached to each film served as a handle and identification of each film. The dose delivered to each film was displayed in the side columns.

**Figure 4:**
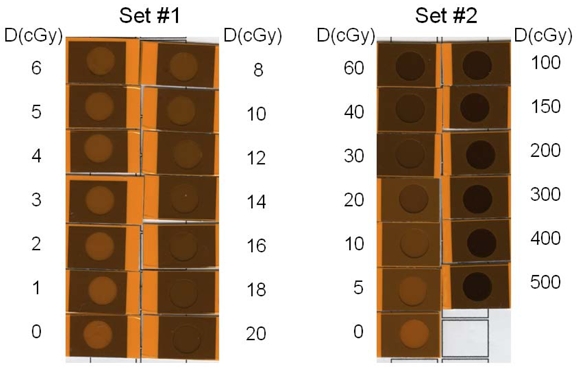
Scanned images of calibration LD cards in two separate sets. Set #1 (left) cards were irradiated with doses from 0 to 20 cGy. Set #2 cards (right) were for doses up to 500 cGy. The dose delivered to each card was displayed in the side columns.

The EBT calibration films (Figure 2, each in a size of 3×3 cm^2^) were irradiated, one at a time, by 6 MV photon beams of a Varian Clinac-2100CD at the center of 10×10 cm^2^ field at d_max_ (1.5 cm) and 100 cm SSD in a Solid Water (RMI457, Gammex, Middleton, WI) phantom (30×30×20 cm^3^). Calibration film doses were calibrated against the ion chamber (with ADCL calibration) measurement at the same location and depth. With the monitor unit settings from 20 through 5000 MU, the doses to the EBT calibration films ranged from 20 to 5000 cGy. The LD calibration films (Figure 3) and cards (Figure 4) were irradiated, one at a time, by 6 MV photon beams of the Clinac-iX at the center of 10×10 cm^2^ field at d_max_ (1.5 cm) and 100 cm SAD in a polystyrene phantom (25×25×15 cm^3^). Calibration film doses were calibrated against the ion chamber (Standard Imaging Exradin A-12 0.65cc thimble chamber with ADCL calibration) measurement at the same location and depth. The output of the treatment machines was calibrated per AAPM TG-51 protocol. With the monitor unit settings from 1 through 500 MU, the doses to the LD calibration films and cards ranged from 1 to 500 cGy.

### IRRADIATION OF EXPERIMENTAL FILMS

**Figure 5:**
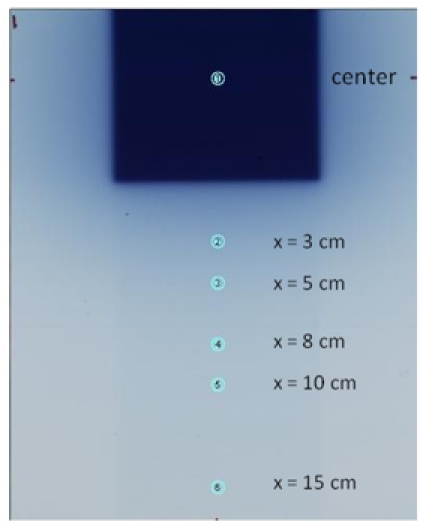
Scanned image of an irradiated experimental EBT film at dmax in a Solid Water phantom. Circular regions of interest of 6 mm diameter centered on the peripheral measurement points were indicated by the circles labeled as 2 through 6.

A full sheet of experimental EBT film was exposed to cover the primary field (10×10 cm^2^) and peripheral region (Figure 5) with 5000 MU at depth of 0, 0.5 cm or d_max_ for 6 and 15MV of the Clinac-iX with 100 cm SAD. The nominal d_max_ values for 6MV and 15MV beams were 1.5 cm and 3.0 cm, respectively. The long edge (25 cm) of the EBT film was parallel to the central line of the cross plane. The authors used two separate phantom configurations: Solid Water (RMI457) and Solid Water with Superflab bolus (Mick RadioNuclear, Mount Vernon, NY) above the EBT film sheet.

**Figure 6:**
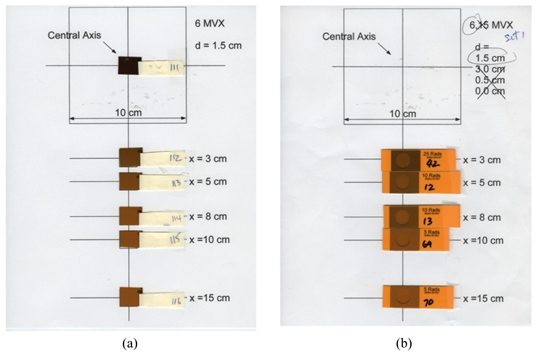
Scanned images of experimental (a) LD films, and (b) LD cards irradiated in peripheral region. The variation of the color shade with the distance from the field edge can be visually distinguished.

For each depth of 0, 0.5 cm or dmax, five experimental LD films or cards were individually positioned on a paper template at the five peripheral points at x = 3, 5, 8, 10 and 15 cm from the 10×10 cm^2^ field edge (Figure 6) in the cross-plane direction. Each set of five experimental LD films or cards were simultaneously exposed by a 6MV or 15MV beam of the Clinac-iX at 100 cm SAD with 500 MU. Polystyrene phantom was used to support the experimental LD films and cards, with Superflab bolus above the films/cards. Two or three repetitive runs for each energy modality were performed for the experimental LD films and cards for statistical reproducibility.

### scanning of films

All experimental, calibration and background LD films and cards were scanned at the same location and orientation of an Epson Perfection 4870 flatbed scanner with reflection mode. All the EBT films were scanned at the same location and orientation of an Epson 10000XL flatbed scanner with transmission mode, because the 10000XL scanner has larger scanner area for the full EBT film sheet. The settings of 48 bit color and 150 dpi were used, color correction was disabled, and files were saved in TIFF format for both scanners. In addition, an X-Rite spot densitometer (with 2 mm aperture) was used to manually read the LD films, one at a time, in the red color channel. The reading and scanning of all the films occurred at least one day after the irradiation.

The calibration and background EBT films were grouped in strips (Figure 2), and each strip was scanned at the scanner bed center, one strip at a time. The calibration and background LD films (cards) were grouped together and scanned (Figures 3 and 4) at the scanner bed center. The full sheet experimental EBT film was scanned one at a time (Figure 5). Each set of five experimental LD films/cards pasted on a paper template were scanned together, one paper sheet at a time, as shown in Figure 6.

### Data processing and analysis

Film data processing was done using a public domain software ImageJ [41] v1.38. (National Institute of Health, Bethesda, MD, http://rsb.info.nih.gov/ij/). Red channel data were extracted and processed. For each calibration or background LD film/card, the average of the pixel values (PV) at the pixels in a circular area of about 6 mm diameter at the film center was calculated and assigned as the PV of the film. For each calibration or background EBT film, the average PV in a square area of 0.5×0.5 cm^2^ at the film center was calculated and assigned as the PV of the film. The PV in the processed image file were converted to optical density (OD), defined by the following equation.

(1) $$OD=\ln\left(\frac{65535}{PV}\right)$$

The OD values from all background films were then averaged to yield the value of OD_background_. The net optical density (NOD) of a calibration film was then calculated by subtracting the OD_background_ from the OD value of the film.

(2) $$NOD=OD-OD_{background}$$

The calibration curve was formed by plotting the NOD values of the calibration films against dose values (in cGy). The calibration curve for red channel was established from the calibration film data for each combination of film/dosimeter cards and densitometer/scanner. Each calibration curve was fitted by a polynomial function, Eq. (3), using TableCurve2D version 5.01.05 software (Systat Software Inc., Chicago, IL).

(3) $$D\left(\text{cGy}\right)=a+b\cdot \left(NOD\right)+c\cdot {\left(NOD\right)}^{d}$$

For each experimental LD film/card, the average PV in a 6 mm circular area centered at the measurement point was assigned as the PV of the film/card. For each experimental EBT film sheet, the average PV values in the 6 mm circular regions of interest centered at the peripheral measurement locations at x = 3, 5, 8, 10, and 15 cm from the field edge (Figure 5) were also calculated and assigned as the PV of those locations. The PV data at the peripheral measurement locations were converted to OD using Eq. (1) and to NOD using Eq. (2). The dose conversion from NOD was accomplished for all the experimental films using the polynomial function fit in Eq. (3). The dose values (in units of cGy) at all the peripheral measurement locations were thus obtained.

### Ion chamber measurements

Absolute dose was measured with an Exradin A-12 0.65 cc thimble ion chamber (Standard Imaging, Middleton, WI) that had an ADCL dose-to-water calibration for Co-60 and an air kerma calibration for a 250 kV beam. The latter, when multiplied by the water-to-air mass energy absorption coefficient ratio, is within 0.5% of the Co-60 calibration factor. Therefore, the ratio of charge collected out-of-field to that collected in-field was taken as the ratio of doses. Ion chamber measurements were performed for 10×10 cm^2^ field size of 6 MV and 15 MV photon beams on the Clinac-iX linear accelerator. For both in-field (primary) and out-of-field (peripheral) measurements the chamber was placed in a small water tank (CNMC model WP-3040) at the depths of 0, 0.5 cm or d_max_ with 100 cm SAD. All the ion chamber data were taken with 500 MU setting.

## RESULTS

### Calibration curves

**Figure 7:**
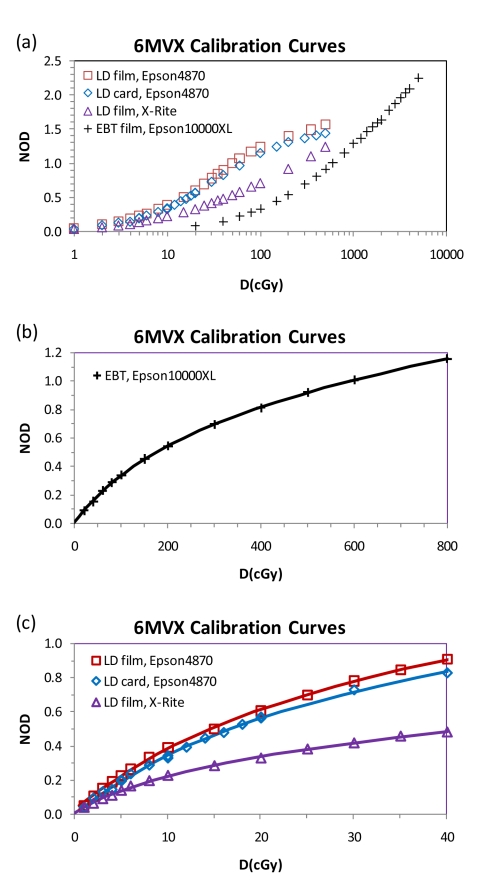
(a) Comparison of the calibration curves with four different combinations of film type and densitometer/scanner for dose up to 5,000 cGy. Note the dose values were plotted in logarithmic scale. (b) Calibration curve for EBT scanned with Epson 10000XL scanner in the dose range up to 800 cGy. The scatter symbols were for the data. The solid curve represented the polynomial fit. (c) Calibration curves for LD film/card in the dose range up to 40 cGy. The scatter symbols were for the data. The solid curves were for the polynomial fit functions.

Due to the weak variation of EBT film response versus photon energy [25, 35], the dose-to-optical density calibration was done for the 6 MV beam only. The NOD values of the calibration EBT, LD films and LD cards were plotted against dose values, as shown in Figures 7a-7c For comparison of the relative sensitivities of the EBT, LD film, and LD cards, the calibration curves in the full dose range up to 5,000 cGy were plotted in Figure 7a, with the dose values in logarithmic scale. This graph demonstrated that the LD sensitivity was about 10 times higher than the EBT film.

To focus on the dose range in the peripheral region, the authors fitted the calibration data to polynomial functions in the dose ranges up to 40 cGy and 800 cGy for LD and EBT, respectively (figure 7b and 7c). The fitting coefficients of the polynomial function (Eq. (3)) for the calibration curves were listed in Table 1 for the four different combinations of film type and densitometer/scanner.

**Table 1:** Fitting coefficients of the polynomial functions for the calibration curves.

**Film type**	**Densitometer/Scanner**	**Fitting coefficients**				**Dose range for fitting**
		**a**	**b**	**c**	**d**	
LD film	X-Rite	-0.21	30.7	166.1	2.6	1 - 40 cGy
LD film	Epson 4870	-0.74	24.4	24.9	3.0	1 - 40 cGy
LD card	Epson 4870	-0.10	25.5	32.4	3.0	1 - 40 cGy
EBT film	Epson 10000XL	-0.02	2.5	3.4	2.7	20 - 800 cGy

### Dose values in phantom

**Figure 8:**
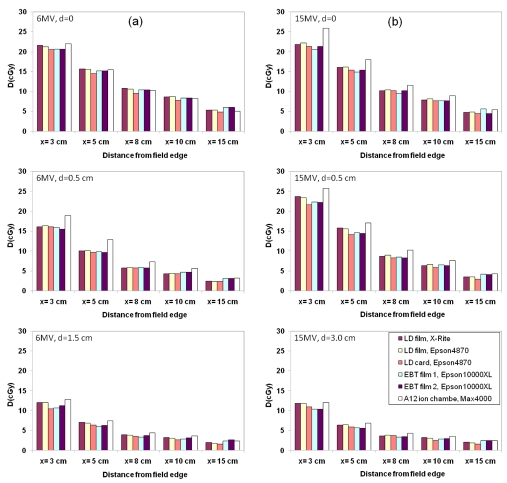
Dose comparisons among EBT, LD films, and LD cards at off-axis distances. (a) 6MV, and (b) 15MV. The legends in the lower graph in (b) apply to all the graphs.

The doses at the peripheral points were determined from the experimental films, and compared with those obtained using ion chamber at respective locations in liquid water phantom. For easier comparison, the EBT measured doses with 5000 MU were normalized to those with 500 MU. Table 2a and Table 2b listed the normalized measured doses with 500 MU in phantoms (EBT film, LD film and LD card) for 6 MV and 15 MV, respectively. Bar graphs showing the dose comparisons were shown in Figure 8. General agreement was found.

**Table 2:** Measured dose values (normalized to 500 MU setting) at various peripheral locations obtained using LD films, LD cards, and EBT films, compared with those measured using thimble ion chamber. (a) for 6MV and (b) for 15 MV photon beams. (a) 6MV Photon Beam

**Detector**	**LD Film**	**LD Film**	**LD Card**	**EBT film 1**	**EBT film 2**	**Ion Chamber**
**Phantom**	**PolystyreneSuperflab**	**PolystyreneSuperflab**	**PolystyreneSuperflab**	**Solid WaterSolid Water**	**Solid WaterSuperflab**	**Water Tank**
**Reader**	**X-Rite**	**Epson4870**	**Epson4870**	**Epson10000XL**	**Epson10000XL**	**Max4000**
**d = 0 (nominal)**						
x = 3cm	21.7 ± 0.6	21.2 ± 0.4	20.5 ± 0.2	20.6	20.6	22.0
x = 5cm	15.6 ± 1.0	15.6 ± 0.4	14.5 ± 0.4	15.2	15.2	15.5
x = 8cm	10.8 ± 0.3	10.6 ± 0.3	9.6 ± 0.4	10.5	10.5	10.4
x = 10cm	8.7 ± 0.2	8.8 ± 0.2	7.8 ± 0.4	8.4	8.4	8.3
x = 15cm	5.4 ± 0.2	5.4 ± 0.2	4.9 ± 0.0	6.1	6.1	5.1
**d = 0.5 cm**						
x = 3cm	16.1 ± 0.1	16.4 ± 0.2	16.1 ± 0.1	15.8	15.5	18.9
x = 5cm	10.1 ± 0.2	10.1 ± 0.2	9.7 ± 0.6	9.8	9.6	12.9
x = 8cm	5.8 ± 0.0	5.8 ± 0.3	5.7 ± 0.5	5.8	5.8	7.3
x = 10cm	4.3 ± 0.3	4.4 ± 0.3	4.3 ± 0.4	4.7	4.7	5.6
x = 15cm	2.5 ± 0.2	2.3 ± 0.2	2.3 ± 0.5	3.1	3.1	3.3
**d = 1.5 cm (dmax)**						
x = 3cm	12.0 ± 0.3	12.0 ± 0.4	10.4 ± 0.3	10.7	11.2	12.8
x = 5cm	7.0 ± 0.0	6.8 ± 0.3	6.4 ± 0.4	5.9	6.3	7.4
x = 8cm	3.9 ± 0.3	3.8 ± 0.3	3.5 ± 0.6	3.4	3.7	4.4
x = 10cm	3.2 ± 0.5	3.0 ± 0.4	2.6 ± 0.3	2.8	3.2	3.6
x = 15cm	1.9 ± 0.2	1.8 ± 0.3	1.6 ± 0.3	2.3	2.6	2.4

**Table 2:** (b) 15MV Photon Beam

**Detector**	**LD Film**	**LD Film**	**LD Card**	**EBT film 1**	**EBT film 2**	**Ion Chamber**
**Phantom**	**PolystyreneSuperflab**	**PolystyreneSuperflab**	**PolystyreneSuperflab**	**Solid WaterSolid Water**	**Solid WaterSuperflab**	**Water Tank**
**Reader**	**X-Rite**	**Epson4870**	**Epson4870**	**Epson10000XL**	**Epson10000XL**	**Max4000**
**d = 0 (nominal)**						
x = 3cm	21.8 ± 0.6	22.2 ± 0.8	21.3 ± 0.3	20.6	20.6	25.9
x = 5cm	16.1 ± 0.4	16.2 ± 0.4	15.4 ± 0.9	14.9	14.9	18.1
x = 8cm	10.2 ± 0.4	10.4 ± 0.3	10.2 ± 0.6	9.5	9.5	11.6
x = 10cm	7.9 ± 0.4	8.2 ± 0.3	7.7 ± 0.3	7.7	7.7	9.0
x = 15cm	4.8 ± 0.2	4.9 ± 0.1	4.5 ± 0.4	5.6	5.6	5.4
**d = 0.5 cm**						
x = 3cm	23.7 ± 1.0	23.4 ± 0.4	21.7 ± 0.9	22.3	22.2	25.7
x = 5cm	15.8 ± 0.8	15.6 ± 0.5	14.1 ± 0.3	14.6	14.4	17.1
x = 8cm	8.7 ± 0.8	9.0 ± 0.4	8.3 ± 0.8	8.5	8.3	10.2
x = 10cm	6.3 ± 0.4	6.6 ± 0.3	5.9 ± 0.0	6.5	6.3	7.6
x = 15cm	3.5 ± 0.0	3.5 ± 0.1	3.0 ± 0.2	4.2	4.1	4.3
**d = 3 cm (dmax)**						
x = 3cm	11.8 ± 0.5	11.8 ± 0.3	10.9 ± 0.4	10.3	10.4	12.1
x = 5cm	6.4 ± 0.3	6.5 ± 0.1	5.8 ± 0.4	5.6	5.6	6.8
x = 8cm	3.6 ± 0.2	3.8 ± 0.3	3.7 ± 0.5	3.3	3.4	4.3
x = 10cm	3.2 ± 0.3	3.0 ± 0.4	2.5 ± 0.4	2.9	2.9	3.5
x = 15cm	2.0 ± 0.2	1.9 ± 0.3	1.5 ± 0.3	2.4	2.5	2.4

## DISCUSSION

The variation of out-of-field (peripheral) dose with distance from field edge has been studied as a function of depth. The technical details concerning the effect of various factors have been discussed [2]. The most important variable is the distance from the edge of the radiation beam. The out-of-field dose diminishes approximately exponentially with distance. The relative dose decreases initially with depth, reaching a minimum at the depth of central ray peak dose d_max_. These characteristics are in agreement with results found by others [2, 42]. Studies showed that the out-of-field dose is qualitatively similar in behavior and of the same order of magnitude for all the treatment machines studied [2, 43-45]. The scatter radiation outside the field edge consists mainly of low-energy photons and electron contamination.

Although pacemakers or ICDs are generally out of the radiation field, it is desirable to know the dose they receive. This is usually measured with TLD, diodes, or OSLD that are calibrated in-field. However, lower energy scattered photons are a more significant part of the out-of-field spectrum than they are of the in-field spectrum. This is shown in Monte Carlo calculations for a 6 MV beam for which the out-of-field spectrum peaks at about 200 keV at 0.1 cm depth [46]. These lower energy photons, combined with a possible energy dependence of the dosimeters, can result in erroneous dose estimates outside the field if in-field calibrations are used. Additionally, the response of dosimeters to the same dose-to-water may vary with distance outside the field as a result of the changing photon spectrum. The response of EBT films was known to be weakly dependent on photon energy across a wide range down to 50 kVp [25, 34, 35], and electron energy in the Megavoltage range [27]. The photon and contaminant electron energies near the surface at the central axis and in the peripheral region have been reported by Edwards [46] and Ding [47]. The effective energies near the surface within the primary field remained close to those at d_max_ [47]. The effective energies in the peripheral region were about 0.3 MeV and above for photons and about 1 MeV and above for contaminant electrons [46, 47]. In the energy range of interest, the EBT film response was reasonably flat within the uncertainty of its estimate [25, 27, 34, 35]. Hence, the authors did not apply an energy correction factor on the data obtained in this study. The reported weak variation of EBT film response with energy was also the reason why a separate dose-to-optical density calibration curve was not done for the 15 MV beam.

Accuracy requirement within 16% in the dose range of out-of-field measurements has been proposed by Kry *et al*. [13, 14]. In this study, it was observed that there were larger percentage differences between the film and ion chamber data for larger distances from the field edge. Nevertheless, the doses at x = 15 cm were less than 1% of the dose at d_max_ along the central axis. Hence, the film and ion chamber data can be considered comparable in practical sense.

The LD films studied in this work were made with similar active emulsion material to that in the EBT films, except that the emulsion layer was much thicker in the LD films. Hence, the energy dependence of the LD film response is expected to be weak, similar to the EBT films. On the other hand, the lamination structure of the LD films was not translucent, different from the EBT films. The lamination layers sandwiching the emulsion material in the LD films were white opaque polyester on one side and yellow transparent polyester on the opposite side. Thus, it is important to use the reflective mode to scan the LD films. The influence of the optical density readings by the LD film orientation on the scanner bed was not investigated in this work. The authors made sure that the film orientations of all LD film pieces were the same on the scanner bed in this study. The LD card was made by further sandwiching LD film by a lamination layer with printed reference-color corresponding to a specific radiation dose surrounding a circular window displaying active LD film, as shown in Figure 1. Hence, the optical densities of the LD film and LD card were slightly different for the same radiation dose, as shown in Figure 7.

The flatbed scanners, Epson models 4870 and 10000XL, were capable of scanning in both transmission and reflection modes. The choices of transmission mode for EBT films and refection mode for LD films and cards were related to the translucence of the EBT films and opaque lamination of the LD film and cards, respectively. All the scans were done with 48-bit color setting. The scanned data files in TIFF format consisted of three parts, 16-bit in each of the red, green and blue channels in the spectrum of the optical sources. The green channel data were also extracted and analyzed, in addition to the red channel data reported here. Similar dose results were obtained from the green channel data. The authors decided to report the doses from red channel data, which were less noise. The X-Rite spot densitometer was also used for LD film data acquisition, for comparison with the flatbed scanner. The light source intensity through the 2-mm aperture in the X-Rite densitometer was actually high enough to penetrate through the LD film allowing for reasonable optical density readings in both red channel and visual (combined color) channel. In this paper, the authors decided to report the red channel data from the scanners and spot densitometer.

The variation of the color shades of the LD films and the circular windows in the LD cards with the distance from the field edge can be visualized immediately after the irradiation was completed (Figure 6). The LD card had visual aid around the sensitive film region (circular window) so that it can allow a quick visual estimate of dose. The printed area surrounding the circular window on the LD card can have certain color scales corresponding to a specific dose, such as 5, 10 or 25 cGy (see Figure 6b). Rough estimation of the dose is possible by reading the color in the circular window and comparing with the referenced color scale surrounding the circle. For example, in Figure 6b, the LD cards at x = 10 cm and 15 cm were labeled as “5 Rads”, because the area surrounding the circular window was printed with the reference color corresponding to the shade of an LD film receiving 5 cGy. Visual evaluation indicated that the doses at x = 10 and 15 cm would be lower than 5 cGy because the color shades in these two circular windows were lighter than the surrounding reference color calibrated for 5 cGy. As listed in Table 2a, the doses determined from the quantitative analysis based on the scanned images were 2.6 and 1.6 cGy at x = 10 and 15 cm, respectively, confirming the visual estimates. Similarly, the circular windows in the LD cards at x = 5 and 8 cm were lighter than the surrounding reference color labeled as “10 Rads”. This led to the visual dose estimates at x = 5 and 8 cm as lower than 10 cGy, also consistent with the quantitative dose determination of 6.4 and 3.5 cGy (see Table 2a).

The LD dosimeter cards can potentially be used as in vivo dosimeters to determine dose to a pacemaker or other critical organs as is frequently done with LiF TLD. The advantage of this LD radiochromic film dosimeter over TLD is its greater ease of use and nearly real time result. After the initial calibration against a standard such as a calibrated ionization chamber, the dosimeters require very little additional effort and are easy to use. In addition, LD cards are inexpensive, accurate, fast, and semi-quantitative before scanning.

## CONCLUSION

It is feasible to accurately measure peripheral doses of megavoltage photon beams using new high-sensitivity radiochromic devices, including GAFCHROMIC® EBT film, Low Dose film and Low Dose cards. This nearly real-time and inexpensive method can be applied in a clinical setting for dose measurement to critical organs and sensitive patient implant devices.
